# Estimation of mean systemic filling pressure in postoperative cardiac surgery patients with three methods

**DOI:** 10.1007/s00134-012-2586-0

**Published:** 2012-05-15

**Authors:** Jacinta J. Maas, Michael R. Pinsky, Bart F. Geerts, Rob B. de Wilde, Jos R. Jansen

**Affiliations:** 1 Department of Intensive Care Medicine, Leiden University Medical Center, PO Box 9600, 2300 RC Leiden, The Netherlands; 2 Department of Critical Care Medicine, University of Pittsburgh, Pittsburgh, PA USA; 3 Department of Anesthesiology, Leiden University Medical Center, Leiden, The Netherlands

**Keywords:** Guyton, Venous return, Mean systemic filling pressure, Cardiac output, Circulation

## Abstract

**Purpose:**

To assess the level of agreement between different bedside estimates of effective circulating blood volume—mean systemic filling pressure (Pmsf), arm equilibrium pressure (Parm) and model analog (Pmsa)—in ICU patients.

**Methods:**

Eleven mechanically ventilated postoperative cardiac surgery patients were studied. Sequential measures were made in the supine position, rotating the bed to a 30° head-up tilt and after fluid loading (500 ml colloid). During each condition four inspiratory hold maneuvers were done to determine Pmsf; arm stop-flow was created by inflating a cuff around the upper arm for 30 s to measure Parm, and Pmsa was estimated from a Guytonian model of the systemic circulation.

**Results:**

Mean Pmsf, Parm and Pmsa across all three states were 20.9 ± 5.6, 19.8 ± 5.7 and 14.9 ± 4.0 mmHg, respectively. Bland-Altman analysis for the difference between Parm and Pmsf showed a non-significant bias of −1.0 ± 3.08 mmHg (*p* = 0.062), a coefficient of variation (COV) of 15 %, and limits of agreement (LOA) of −7.3 and 5.2 mmHg. For the difference between Pmsf and Pmsa we found a bias of −6.0 ± 3.1 mmHg (*p* < 0.001), COV 17 % and LOA −12.4 and 0.3 mmHg. Changes in Pmsf and Parm and in Pmsf and Pmsa were directionally concordant in response to head-up tilt and volume loading.

**Conclusions:**

Parm and Pmsf are interchangeable in mechanically ventilated postoperative cardiac surgery patients. Changes in effective circulatory volume are tracked well by changes in Parm and Pmsa.

## Introduction

Accurate assessment of the cardiovascular state in the critically ill is difficult because easily measured parameters, such as blood pressure and cardiac output (CO), can co-exist with different levels of ventricular pump function and effective circulating blood volume. Thus, identifying the appropriate therapy and targeting specific measurable endpoints of therapy are problematic. Although assessing dynamic changes in arterial pulse pressure or left ventricular stroke volume during ventilation and passive leg-raising maneuvers improves identification of fluid responsiveness, they do not quantify the effective circulating blood volume, or the cause or lack thereof. Although fluid resuscitation therapy is important in the management of unstable patients, excessive fluid resuscitation can be harmful in acute lung injury [1], head injury [2] and postoperative patients [3]. Thus, a measure of effective volume status is useful to avoid volume overload since even volume-overloaded patients may remain volume responsive.

Mean systemic filling pressure (Pmsf) is a functional measure of the effective intravascular volume status. It is the pressure anywhere in the circulation during circulatory arrest [4]. Importantly, the central venous pressure (Pcv) to Pmsf pressure difference defines the driving pressure for venous return, and together with the resistance to venous return defines CO. We have shown that Pmsf can be measured in ventilator-dependent patients using inspiratory-hold maneuvers defining Pcv-CO data pairs that when extrapolated to zero CO reports Pmsf [5, 6]. This calculated Pmsf parameter accurately follows changes in intravascular volume [5, 7].

Unfortunately, this inspiratory-hold technique requires a sedated and ventilated patient, not universally seen in critically ill patients. We thus studied two simpler bedside methods for determining Pmsf as previously suggested by Anderson [8] and Parkin [9]. Anderson hypothesized that the circulation of the arm behaves similarly to total systemic circulation during steady state conditions. Accordingly, we measured transient stop-flow forearm arterial and venous equilibrium pressure, referred to as arm equilibrium pressure (Parm). Parkin [9] proposed estimating the effective circulatory volume based on an electrical analog simplification of Guytonian circulatory physiology estimating the mean circulatory pressure (Pmsa) from directly measured Pcv, mean arterial pressure and CO. The aim of our study was to compare the level of agreement among simultaneously measured Pmsf, Parm and Pmsa in three intravascular volume states in critically ill patients.

## Materials and methods

The study was approved by the hospital ethics committee of Leiden University Medical Center (P01.111, 29 January 2002) and carried out in Leiden. Written informed consent was obtained from all patients prior to surgery. The institutional review board of the University of Pittsburgh approved the review and analysis of data. Eleven patients were enrolled and studied after cardiac surgery.

### Patients

We limited our study to cardiac surgery patients requiring pulmonary artery and radial artery catheters for perioperative monitoring. Our study partially used hemodynamic data from the same patients reported in another study, but examined different protocol-based measures [7]. All patients had coronary artery or valvular disease with preserved ventricular function (EFlv > 0.4). Patients with aortic aneurysm, severe peripheral vascular disease, postoperative arrhythmia, postoperative valvular insufficiency or needing artificial pacing or the use of a cardiac assist device were excluded. All subjects were studied during their initial postoperative period in the ICU while sedated (propofol 3.0 mg kg^−1^ h^−1^ and sufentanil 0.06–0.19 µg kg^−1^ h^−1^) and mechanically ventilated with airway pressure release ventilation adjusted to achieve normocapnia, with 7–11 ml kg^−1^ tidal volumes, 5 cmH_2_O positive end-expiratory pressure, FiO_2_ 0.4 and *f* = 11–13 min^−1^ (Evita 4, Dräger AG, Lübeck, Germany). During the study interval all subjects were hemodynamically stable and no changes were made in their vasoactive drug therapy.

### Measurements

All subjects also had a central venous catheter. Arterial pressure (Pa) and Pcv were recorded on a computer for off-line analysis. Pa and Pcv pressure transducers were referenced to the intersection of the anterior axillar line and the 5th intercostal space, and re-referenced after a 30° head-up rotation. Airway pressure (Paw) was measured at the proximal end of the endotracheal tube. Beat-to-beat cardiac output (CO) was obtained by Modelflow pulse contour analysis as previously described by us [10–12]. We calibrated the pulse contour CO measurements with three thermodilution CO measurements equally spread over the ventilatory cycle [11].

We have previously described the inspiratory-hold method for estimating Pmsf [5]. Briefly, four 12-s inspiratory holds were applied at Paw of 5, 15, 25 and 35 cmH_2_O, respectively. The resulting Pcv and CO were measured during the plateau phase (between 7 and 12 s of each inspiratory hold maneuver), and the zero CO intercept of the Pcv and CO pairs estimated Pmsf.

**Fig. 1:**
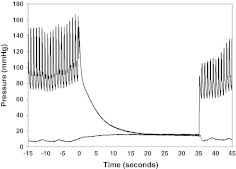
Representative radial artery pressure and venous pressure trends before (−15 to 0 s), during (0 to 35 s) and after the occlusion of the upper arm of a patient. Arm vascular occlusion equilibrium pressure (Parm) is taken as the arterial pressure 30 s after stop-flow. Note the influence of mechanical ventilation on arterial and venous pressure before and after occlusion

Parm estimates of Pmsf [8] assumes Pa and Pv equilibrium following rapid vascular occlusion. We performed a pilot study in nine patients after either cardiac surgery or cardiopulmonary resuscitation to determine the stop-flow time. We measured arterial and venous pressures in the same hand and created upper extremity blood stop-flow using a rapid cuff inflator (Hokanson E20, Bellevue, Washington) to pressures 50 mmHg above systolic pressure and held occlusion for 35–60 s (Fig. 1). Measurements were performed three times to assess repeatability (Table 1). Arterial and venous pressures equilibrated after 25–30 s of stop-flow, with a mean difference of −0.73 ± 1.07 mmHg at 30 s. Thus, we chose the 30-s value of the arterial pressure for Parm for the present study.

**Table 1:** Pilot study arm equilibrium pressure

Time (s)	Pa			Pv			Pa–Pv		
	Mean (mmHg)	SD (mmHg)	Repeat (%)	Mean (mmHg)	SD (mmHg)	Repeat (%)	Mean (mmHg)	SD (mmHg)	Repeat (%)
15	23.32	2.41	5.45	21.96	2.05	9.20	1.35	2.69	4.89
20	22.11	1.88	6.11	22.12	2.02	9.58	−0.01	1.62	5.52
25	21.42	1.56	6.91	22.06	1.91	9.79	−0.63	1.02	5.18
30	21.08	1.38	6.55	21.81	2.05	9.58	−0.73	1.07	4.55

Effect of time on radial arterial pressure (Pa), peripheral venous pressure (Pv) and the difference between Pa and Pv during upper arm stop-flow. The results of a pilot study in nine patients are indicated. Repeat, the averaged repeatability of three sequential measurements

*SD* Standard deviation

The Pmsa estimate [9] uses a mathematical model of the systemic circulation comprising compliant arterial and venous compartments and resistances to blood flow. The model parameters are adjusted to match those of the patient’s current measured variables, such that Pmsa = *a* × Pcv + *b* × Pa + *c* × CO, where *a* and *b* are dimensionless constants (*a* + *b* = 1, typically *a* = 0.96, *b* = 0.04), and c has the dimensions of resistance and is a function of the patient’s height, weight and age.

*c* = 0.038 × (94.17 + 0.193 × age)**/**(4.5 × [0.9^(age−15)^] × 0.007184 × [height^0.725^] × [weight^0.425^]).

### Protocol

Measurements were carried out within 2 h of arrival in the ICU following initial hemodynamic stabilization. To induce changes in volume status, measurements were performed in the supine position (baseline), in a 30° head-up tilt (HUT) and again in the supine position after 500 ml hydroxyethyl starch (HES 130/0.4) rapid fluid administration (VOL). Measurements of Pa, Pv, Pcv and CO were done during baseline in the supine position, 2 min after the change to HUT and 2–5 min after fluid loading, with Pmsf, Parm and Pmsa calculated for each step. Repeatability of Parm was determined by two measurements at baseline and after VOL. The study protocol lasted about 60 min. All patients completed all steps of the protocol, and there were no adverse events.

### Statistical analysis

After confirming normal distribution of data with the Kolmogorov-Smirnov test, differences among Pmsf, Parm and Pmsa during baseline, HUT and VOL were analyzed using paired *t* tests. Calculations of bias, precision and limits of agreement (LOA) between Pmsf and both Parm and Pmsa were performed using Bland-Altman analysis with bias reflecting the mean difference between Pmsf and either Parm or Pmsa and precision as the standard deviation (SD) of these differences. After adjustment for the number of observations (*n* = 33), LOA were defined as bias ± 2.04 × SD. For repeatability of Parm (*n* = 40) LOAs were bias ± 2.02 × SD. The coefficient of variation (COV) was calculated as 100 % × SD/mean. Repeatability of Parm was calculated by Bland-Altman analysis using duplicate measurements at baseline and after VOL, which were pooled together. A *p* value <0.05 was considered statistically significant. Unless otherwise stated, data are presented as mean ± SD.

## Results

Patient characteristics are presented in Table 2 and mean hemodynamic data for the protocol in Table 3. Mean Pa decreased during HUT and was unchanged with VOL. Pcv, CO, Pmsf, Parm and Pmsa decreased during HUT and increased with VOL.

**Table 2:** Patient characteristics

	Mean	Range
Age (years)	64	50–80
Gender	9 male, 2 female	
Weight (kg)	86	73–112
Length (cm)	174	158–190
Surgery		
CABG	9	
AVR	2	
Respiratory rate (min^−1^)	12	11–13
Tidal volume/predicted (ml kg^−1^)	9	7–11
PEEP (cmH_2_O)	5	

	Number of patients	Range dose (µg kg^−1^ min^−1^)
Vasoactive medication		
Dobutamine	4	2–4
Enoximone	1	2
Norepinephrine	5	0.01–0.09
Sodium nitroprusside	1	0.25

*CABG* Coronary artery bypass grafting, *AVR* aortic valve replacement

**Table 3:** Hemodynamic data of patients during baseline, HUT and VOL

	Baseline		HUT			VOL		
	Mean	SD	Mean	SD	p1	Mean	SD	p2
Pa (mmHg)	88.8	17.9	77.3	17.0	<0.001	97.9	15.3	0.003
Psys (mmHg)	128.5	21.9	107.2	16.9	0.001	143.3	17.7	0.004
Pdias (mmHg)	69.0	17.7	62.4	17.9	0.001	75.2	15.6	0.040
PP (mmHg)	59.5	14.7	44.8	9.9	0.016	68.1	12.1	0.076
Pcv (mmHg)	7.1	2.0	4.4	1.8	0.001	10.4	1.3	0.001
CO (l min^−1^)	5.8	1.6	4.8	1.2	0.006	7.0	1.7	0.004
HR (min^−1^)	88	14	87	15	0.574	86	10	0.475
Pmsf (mmHg)	19.7	3.9	16.2	3.0	0.001	28.3	3.6	<0.001
Parm (mmHg)	18.4	3.7	15.4	3.1	0.001	27.1	4.0	<0.001
Pmsa (mmHg)	14.7	2.7	10.9	2.0	<0.001	19.2	1.1	<0.001

Values are mean ± SD; *n* = 11 patients

*HUT* Head-up tilt, *VOL* after volume loading (+500 ml), *Pa* arterial pressure, *Psys* systolic arterial pressure, *Pdias* diastolic arterial pressure, *PP* pulse pressure, *Pcv* central venous pressure, *CO* cardiac output, *HR* heart rate, *Pmsf* mean systemic filling pressure, *Parm* arm equilibrium pressure, *Pmsa* model analog mean circulatory pressure

Statistical comparison, p1 and p2, paired *t* test between baseline and HUT and between baseline and VOL

**Fig. 2:**
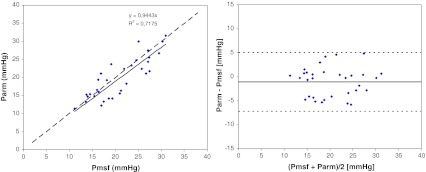
Regression (**a**) and Bland-Altman analysis (**b**) of arm equilibrium pressure (Parm) and mean systemic filling pressure (Pmsf). In **a**, the *solid line* is the regression line, and the *dashed line* is the line of identity. In **b**, the *solid line* indicates the bias, and the *dashed lines* are the limits of agreement

Pmsf, Parm and Pmsa decreased in all patients during HUT (3.4 ± 2.6, 3.0 ± 2.0 and 3.7 ± 2.3 mmHg, *p* < 0.001, *p* = 0.001, respectively). VOL was associated with an increase in Pmsf, Parm and Pmsa (8.7 ± 5.3, 8.7 ± 3.8 and 4.5 ± 2.1 mmHg, *p* < 0.001 all, respectively). Parm was not different from the Pmsf during the baseline, HUT or VOL (*p* = 0.236, *p* = 0.423 and *p* = 0.173, respectively). However, Pmsf and Pmsa differed significantly for the three conditions (*p* < 0.001 all). Pmsf regressed significantly with Parm (Fig. 2a) [slope = 0.944, correlation coefficient (*R*) = 0.847] and Pmsa (Fig. 2b) (slope = 0.704, *R* = 0.822).

Baseline Pmsf and Parm did not correlate with Pcv, Pa and pulse pressure. Baseline Pmsa correlated with Pcv (Pearson's correlation coefficient *R* = 0.846, *p* = 0.001) and with pulse pressure (*R* = 0.697, *p* = 0.017). Pmsa did not correlate with mean, systolic and diastolic arterial pressure (*p* > 0.28 for all).

For the changes in Pmsf, Parm and Pmsa induced by HUT, only Pmsa correlated significantly with changes in Pcv (*R* = 0.931, *p* < 0.001). For the changes induced by VOL both Pmsf and Pmsa correlated with changes in Pcv (*R* = 0.781, *p* = 0.005 and *R* = 0.911, *p* < 0.001). No significant correlation was found with changes in Pa or pulse pressure for changes in Pmsf, Parm and Pmsa.

### Agreement of methods

**Fig. 3:**
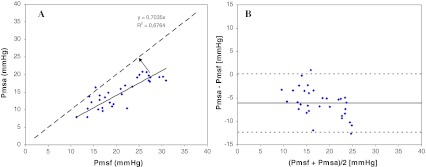
Regression (**a**) and Bland-Altman analysis (**b**) of model analog pressure (Pmsa) and mean systemic filling pressure (Pmsf). In **a**, the *solid line* is the regression line, and the *dashed line* is the line of identity. In **b**, the *solid line* indicates the bias, and the *dashed lines* are the limits of agreement

For all measurements Pmsf and Parm displayed a non-significant bias of −1.0 ± 3.08 mmHg (*p* = 0.062), COV of 15 % and with LOA of −7.3 and 5.2 mmHg (Fig. 2b). The biases for Pmsf and Parm were: baseline −1.3 ± 3.4, HUT −0.8 ± 3.2, VOL −1.2 ± 2.8 mmHg. For all measurements Pmsf and Pmsa displayed a bias of −6.0 ± 3.1 mmHg (*p* < 0.001), COV of 17 % and LOA of −12.4 and 0.3 mmHg (Fig. 3b). The biases for Pmsf and Pmsa were: baseline −5.0 ± 2.8, HUT −5.3 ± 3.2 and VOL −8.1 ± 2.7 mmHg. Mean Pmsf, Parm and Pmsa across all three states were 20.9 ± 5.6, 19.8 ± 5.7 and 14.9 ± 4.0 mmHg, respectively.

**Fig. 4:**
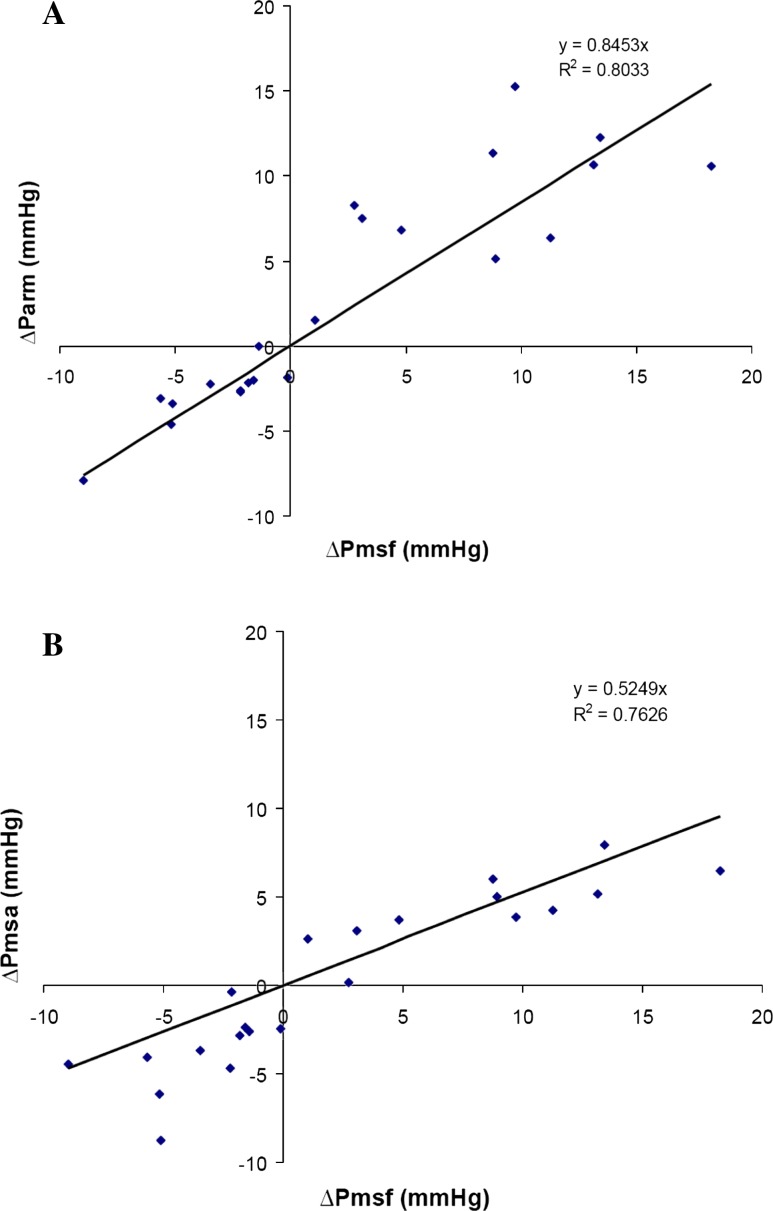
Changes in mean systemic filling pressure by arm equilibrium pressure (ΔParm) (**a**) and by model analog (ΔPmsa) (**b**) plotted against changes in mean systemic filling pressure by inspiratory hold procedures (ΔPmsf). The regression line is indicated by a *solid line*

Changes of Parm (ΔParm) and Pmsa (ΔPmsa) versus changes in Pmsf (ΔPmsf) are shown in Fig. 4. Both ΔParm and ΔPmsa regressed significantly (*p* < 0.001) with ΔPmsf (slope = 0.85, *R* = 0.896 and slope = 0.53, *R* = 0.871, respectively). The cross tabulation agreement of positive and negative changes in each of the methods for HUT and VOL displayed directionally balanced concordance for all data pairs for both ΔParm and ΔPmsa versus ΔPmsf.

### Repeatability of Parm

Bland-Altman analysis for Parm duplicate measurements during both baseline and VOL revealed a bias of 0.03 ± 1.02 mmHg, LOA from −2.04 to 2.09 mmHg and COV of 5 %. No difference was found between the first and second of the duplicate Parm measurements (*p* = 0.915).

## Discussion

Our study demonstrates that estimates of Pmsf measured 30 s after arm stop-flow (Parm) are interchangeable with Pmsf calculated using inspiratory hold maneuvers in mechanically ventilated postoperative cardiac surgery patients. Furthermore, changes in volume status by HUT and VOL are similarly tracked by Pmsf, Parm and Pmsa. These data support the hypothesis formulated, but not previously validated, by Anderson [8], that during steady-state flow conditions the arm is representative of the entire circulation, such that a rapid vascular occlusion will result in its stop-flow Pa approximating Pmsf. Thus, both Pmsf and Parm can be used at the bedside to measure effective circulating blood volume. Furthermore, Pmsa can reliably tract changes in effective circulating blood volume status.

The use of both Parm and Pmsa has practical advantages over our previously validated inspiratory-hold maneuver Pmsf approach. Neither requires positive-pressure breathing or multiple simultaneous measures of Pcv and CO during inspiratory hold maneuvers, and both can be rapidly and repeatedly measured sequentially as treatment or time progresses. Parm requires only the peripheral arterial catheter. Pmsa requires both central venous and peripheral arterial catheters. Thus, these two novel approaches markedly increase the applicability of assessment of effective circulating blood volume to a broader patient population.

### Methodological considerations

#### Radial artery pressure

Shortly after cardiopulmonary bypass, radial artery pressure can be significantly less than aortic pressure [13–15], but this difference disappears after about 60 min, coinciding with hemodynamic stabilization [13]. Our study started after approximately 2 h after cardiopulmonary bypass in stable patients. Therefore, we believe that the mean radial artery pressure reliably reflected the central aortic pressure. We recently documented in a porcine model of acute endotoxemia [16] that similar central-to-regional arterial pulse pressure changes occur. However, the value of Pmsf is not dependent on the calibration of the pulse contour method as long as a linear change in CO is followed by a linear change in CO derived from the pulse contour. Indeed, Pcv at CO equal to zero is not even influenced by a calibration factor.

#### Arm stop-flow procedure

In the pilot stop-flow study described above, we observed that a plateau pressure developed in both arterial and venous pressures after 20–30 s, as predicted by Anderson [8]. However, a further decrement in both Pa and Pv developed after 35–40 s, indicating probable hypoxia-induced vasodilation. We also observed the best repeatability and lowest standard deviations between the arterial and venous pressure at 25–30 s of stop-flow, which was the time we used in this study. Furthermore, stop-flow durations longer than 5 min are needed to produce reactive hyperemia in the human forearm [17, 18]. Thus, if stop-flow maneuvers are limited to <1 min, regional blood flow will also normalize after an additional 1 min [19]. The rapid cuff inflator inflates in less than 0.3 s [20]. In this time there is only a brief cessation of venous return prior to arterial stop-flow equal to approximately one heart beat. We expect this overestimation to be negligible because the amount of inflow is small compared to the total distal arm blood volume. Finally, since longer vascular occlusion maneuvers are routinely used to assess dynamic changes in tissue O_2_ saturation without complications [21], we feel that this much shorter vascular occlusion maneuver is safe.

#### Model analog Pmsa

No clinical evaluation of Pmsa against other methods to measure Pmsf has been done so far. The validity of the Pmsa algorithm was successfully tested using a closed loop control of fluid replacement during continuous hemodiafiltration [22]. Our data support these findings because ΔPmsf and ΔPmsa faithfully track each other.

#### Pmsf

We showed that Modelflow pulse contour CO was interchangeable with pulmonary artery and aortic flow probe-derived CO in swine [23], and that Modelflow-derived Pmsf was interchangeable with flow probe-derived Pmsf with a COV for duplicate measurements of 6.1 %. Still, we report mean baseline Pmsf values of 19.7 mmHg in our cardiac surgical patients, which are higher than the Pmsf values reported between 7 and 12 mmHg in animal studies [24–27]. Using the same inspiratory hold technique and pulse contour analysis, we found Pmsf values of 10.38 ± 1.09 mmHg in pigs [23]. A primary difference between the prior animal studies and our patient observations is the difference in baseline Pcv. In the animal studies this value is close to zero, whereas Pcv in our patient population is 7.1 mmHg on average. The pressure gradient for venous return (Pmsf minus Pcv) in our study (12–13 mmHg) is therefore similar to the pressure gradient for venous return in the animal studies. Thus, our Pmsf values are coupled with the increased Pcv. However, high Pmsf values still predispose patients to peripheral edema formation.

Jellinek et al. [28] and Schipke et al. [29] estimated Pmsf in patients during episodes of apnea and ventricular fibrillation, and found a mean Pmsf value of 10.2 and 12 mmHg, respectively. However, both studies were done on highly anesthetized, non-volume resuscitated subjects. Our method for estimating Pmsf differs considerably from stopping flow by ventricular fibrillation, and our method allows an estimation of Pmsf with intact circulation, applicable in the intensive care unit [5, 30].

### Agreement among Parm, Pmsa and Pmsf

We found agreement among Pmsf and Parm (Fig. 2), and ΔPmsf and ΔParm were concordant in all interventions (Fig. 4). Therefore, both methods should equally measure and follow changes in effective circulating blood volume. There was poor agreement between Pmsa and Pmsf. The large bias makes the methods non-interchangeable. However, the full concordance between ΔPmsf and ΔPmsa indicates that the Pmsa method is very applicable to track changes in effective circulating blood volume, as indeed was documented by Parkin et al. [22] in dialysis patients.

Finally, effective circulating blood volume is a functional measure, not an absolute one. In our study the vasoactive medication was not changed. Changing vasomotor tone will alter unstressed volume, stressed volume and compliance. Any treatment that alters unstressed volume will also alter effective circulating blood volume independent of changes in blood volume, as was demonstrated by Guyton et al. [4].

Can either Parm or Pmsa replace the Pmsf method in the bedside assessment of effective circulating blood volume? Based on the established argument of Critchley and Critchley [31], a new method may replace an older established method if the new method itself has errors not greater than the older method. The Parm method showed a non-significant bias when compared to Pmsf. A single measurement of Pmsf has a COV of about 6 % [23]. We found a 5 % repeatability for Parm. Thus, our data support the argument that Parm may replace inspiratory hold-maneuver generated Pmsf.

A significant bias (*p* < 0.001) was observed between Pmsf and Pmsa, precluding the substitution of raw Pmsa values for Pmsf. However, based on the linearity of Pmsf and Pmsa (Fig. 3a) one can adjust the Pmsa values using a calibration factor of 1.42 (i.e., the reciprocal of the slope of the regression 0.704). After this calibration is applied to Pmsa values, indicated in Fig. 3a by an arrow from the regression line to the line of identity, the bias reduces to zero. The expected precision of the calculation of Pmsa is approximately 10 % (this COV is largely caused by the COV in Pcv measurement; a value of 10 mmHg can be 9.51 or 10.49 mmHg). Although this 10 % is higher than the 6 % for Pmsf, after recalibration, the Pmsa model analog may replace Pmsf. It must be emphasized that the correction factor only describes our postoperative cardiac surgery population and will require similar validation in other patient groups.

### Study limitations

The number of patients included in the study is relatively low. However, we still found a significant difference between Pmsa and Pmsf. With a larger study population the difference between Pmsf and Parm could have become significant. However, the absolute difference of −1.0 mmHg is not clinically relevant. We included patients with preserved left ventricular function after relatively simple cardiac surgery, and excluded patients with previous myocardial infarction and/or congestive heart failure (New York Heart Association class 4). These patients are known to have markedly increased vascular tone with an associated decreased proportional unstressed vascular volume. Thus, caution needs to be used when extrapolating the accuracy of these comparisons to other patients groups. During the study we made no changes in medication. Therefore, we cannot report on the values and comparison of Pmsf, Parm and Pmsa during changes in vasoactive medication, which influences vascular elastance, resistance and conductance properties. A fundamental limitation of the Parm technique is the need to measure arterial pressure from a radial arterial site. In patients with sepsis or on high levels of vasopressors, radial artery compliance may not reflect central arterial compliance, although the mean Pa remains accurate [16]. Therefore, in these patients it is not clear if Parm or Pmsa will tract Pmsf. Still, under these conditions, the diagnosis of decreased effective circulating blood volume is rarely an issue.

## Conclusions

The equilibrium pressure in the arm during stop-flow (Parm) and inspiratory-hold maneuver-derived Pmsf values are interchangeable in mechanically ventilated postoperative cardiac surgery patients. Thus, the mean systemic filling pressure can be simply measured at the bedside by measuring arterial pressure during upper arm stop-flow, without the need for inspiratory hold maneuvers or central venous or pulmonary artery catheters. Furthermore, changes in effective circulatory volume are accurately trended by changes in both Parm and Pmsa.
